# Effects of Yinzhihuang Granules on Serum Liver Enzymes in Jaundice Patients: A Real-World Study Based on HIS Data

**DOI:** 10.1155/2020/3843752

**Published:** 2020-11-05

**Authors:** Cheng Zhang, Lidan Zhang, Jian Lyu, Yanming Xie, Yuting Xie

**Affiliations:** ^1^Institute of Basic Research in Clinical Medicine, China Academy of Chinese Medical Sciences, Beijing 100700, China; ^2^School of Statistics, Renmin University of China, Beijing 100872, China

## Abstract

**Objective:**

Our aim was to analyze the influence of Yinzhihuang granules on serum liver enzymes in jaundice patients and to provide real-world evidence for the efficacy evaluation of Yinzhihuang granules in treating jaundice.

**Methods:**

We constructed a data warehouse which integrates real-world electronic medical records from the hospital information system of multiple 3A hospitals in China and used a descriptive statistical method to analyze the changes in the serum liver enzymes of the jaundice patients treated with Yinzhihuang granules and then used Wilcoxon signed-rank to test the changes in the indicators caused by the treatment.

**Results:**

After being treated with Yinzhihuang granules, the jaundice patients had a decrease in the average serum levels of total bilirubin, indirect bilirubin, aspartate aminotransferase, glutamyl transpeptidase, and alkaline phosphatase, and the differences were statistically significant (*P* < 0.05) but had no significant changes in the average serum levels of direct bilirubin and alanine aminotransferase (*P* > 0.05).

**Conclusion:**

The data analysis on the real-world electronic medical records demonstrate that Yinzhihuang granules help to reduce jaundice patients' serum levels of total bilirubin, indirect bilirubin, aspartate aminotransferase, glutamyl transpeptidase, and alkaline phosphatase, but there is no evidence that Yinzhihuang granules help to reduce the jaundice patients' serum levels of direct bilirubin and alanine aminotransferase.

## 1. Introduction

Jaundice, the yellow staining of the skin, sclera, and mucous membrane caused by the elevation of bilirubin, is a common indicator of hepatobiliary diseases [1]. Jaundice occurs when the plasma bilirubin is excessive or when the damaged liver fails to clear bodily bilirubin [2]. In addition to yellow skin, jaundice patients may suffer from stomachache, nausea, fever, weight loss, or itching [3]. The disease should be treated differently in accordance with the different disease etiology, but the general principle is “to remove jaundice, protect liver, reduce enzyme.” With the verified effectiveness and very few adverse reactions reported, the traditional Chinese medicine (TCM) has certain advantages in treating jaundice. According to TCM, jaundice is a common clinical syndrome primarily indicated by yellow skin, yellow eye, and yellow urine [4] and has multiple different pathogenesis such as dampness, heat, cold, blood stasis, and spleen deficiency, with pathogenic dampness being the dominant one; thus, the treatment should be based on syndrome differentiation. For the patients with damp-heat brewing internally, Zhongjing Zhang wrote in “Treatise on Febrile Diseases” that Yinchenhao decoction can be used to treat them [5].

Yinzhihuang granule, derived from Yinchenhao decoction, is a proprietary Chinese medicine to treat jaundice. Its ingredients are extracted from *Artemisiae Scopariae Herba*, *Gardeniae Fructus*, *Scutellariae Radix*, and *Lonicerae Japonicae Flos*. According to the drug package insert records, it is effective in clearing heat and detoxifying and clearing dampness and removing jaundice. It can be used to treat jaundice caused by liver-gallbladder dampness-heat with the symptoms of yellow skin and eye, chest pain, nausea and vomiting, reddish yellow urine, and the acute or chronic hepatitis with the aforementioned syndromes.

At present, the evaluation of effectiveness of Yinzhihuang granules on jaundice is based primarily on randomized controlled trials; for example, the latest systematic review included nine randomized controlled trials [6]. The evaluation demonstrated that the blue light treatment of neonatal pathologic jaundice, when combined with Yinzhihuang granules administration, was better than the sole blue light treatment, in terms of both clinical effective rate and jaundice fading time (*P* < 0.05). In terms of safety studies, no serious adverse reactions have been reported in the literature, but the instructions of Yinzhihuang granules indicate that there are reports of diarrhea, vomiting, and rash.

The elevation of serum liver enzyme levels is common in liver diseases; the abnormalities of liver enzyme indicators are associated with the possible etiology of liver disease. The enzymes that are commonly detected include alanine aminotransferase (ALT), aspartate aminotransferase (AST), alkaline phosphatase (ALP), *γ*-glutamyl transpeptidase (GGT), and serum bilirubin [7]. These liver enzymes are not only important diagnosis biomarkers but also important indicators of curative effects on, and prognosis of jaundice. The elevation of serum bilirubin levels is the direct cause of jaundice [8]. ALT, present in high concentrations in hepatocyte cytoplasm, is very sensitive to hepatocyte injury and is the most specific traditional biomarker. AST, also present in hepatocytes, can be used to determine whether or not jaundice is caused by liver injury [9], but its liver specificity is relatively low [10]. The elevation of ALP and GGT levels indicate cholestasis. In jaundice patients, the elevation of ALP levels can be used to diagnose obstructive jaundice because it may indicate diffuse hepatocyte dysfunction or severe bile duct obstruction, which obstructs the adequate bile flow [11].

Unlike the traditional randomized controlled trial performed under strict conditions, the real-world study, which can evaluate the actual drug efficacy, is based on the actual medical environment. We performed a retrospective study of hospital electronic medical records data, analyzed the changes in the serum liver enzyme indicators caused by Yinzhihuang granules intervention, and provided the real-world evidences to support Yinzhihuang granules in treating jaundice.

## 2. Materials and Methods

### 2.1. Data Source and Normalization

The data we used were taken from a large-scale data warehouse where electronic medical records are integrated and which was constructed, on the basis of the Chinese 3A hospital information system (HIS), by Institute of Basic Research in Clinical Medicine, China Academy of Chinese Medical Sciences [12]. From the warehouse, we extracted the data of 3610 jaundice patients who had been treated with Yinzhihuang granules and who met the jaundice diagnosis standard: serum total bilirubin (TBil) > 17.1 *μ*mol/L (namely, 1.0 mg/dl) [13]. Their laboratory test information was extracted and analyzed, including seven serum liver enzyme indicators: TBil, direct bilirubin (DBil), indirect bilirubin (IBil), ALT, AST, GGT, and ALP.

Because the data used were from different hospitals in the country and they might adopt different standards for the same project, the investigators, to facilitate analysis [14], normalized the data by converting the units of TBil, DBil, and IBil from mg/dl to *μ*mol/L, which entailed multiplying the value by 17.1 [13].

### 2.2. Inclusion and Exclusion Criteria

The jaundice patients from the HIS database who met the following criteria were included in our study: (1) they were treated with Yinzhihuang granules; (2) their TBil values were greater than 17.1 *μ*mol/L (1.0 mg/dl) for at least one test; (3) there were at least one detection of serum liver enzymes seven days before and seven days after the use of Yinzhihuang granules. On the other hand, those jaundice patients were excluded from the study whose serum liver enzyme detections before and after the medication were both absent.

### 2.3. Data Definition and Outcome Determination

For every included patient, two sets of liver enzyme data were extracted: one set was obtained within seven days before Yinzhihuang granules treatment, and the other set was obtained within seven days after the medication withdrawal. If there were more than one set of data satisfying the condition, then the latest one within seven days before the treatment was defined to be the “pre-medication physicochemical indicator” and the earliest one within seven days after the medication withdrawal was defined to be the “post-medication physicochemical indicator” [15].

The criteria for liver enzyme abnormality were based on each hospital's respective physicochemical reference value. A liver enzyme indicator is abnormal if it is above the upper bound of the normal values. No matter whether the premedication indicators were normal or not, as long as the postmedication indicators were normal, the patient was recorded as “post-medication normal changes.” If both the pre- and postmedication indicators were abnormal but the degree of abnormality decreased after the medication, then the patient was also recorded as “post-medication normal changes.” If the patient was normal before medication but abnormal after the medication, or abnormal before the medication and more abnormal after the medication, then the patient was recorded as “post-medication abnormal changes” [16].

### 2.4. Statistical Analysis Methods and Software

All the data analyses were performed by the software SAS 9.4. We did the descriptive statistics and quantitative analysis on the general data of the jaundice patients' serum liver enzymes tested before and after the medication. For the normally distributed variables, the mean, standard deviation, median value, maximum value, and minimum value were obtained. For the nonnormally distributed variables, the median value, interquartile range, maximum value, and minimum value were obtained. Qualitative analysis was performed on the normal/abnormal changes of the jaundice patients' liver enzymes caused by the medication; namely, the postmedication live enzyme values were compared to the premedication live enzyme values. When the difference between the two datasets followed the normal distribution, it was tested by the paired sample *t*-test. When the difference did not follow the normal distribution, it was tested by the nonparametric test (Wilcoxon signed-rank). *P* < 0.05 was considered statistically significant.

## 3. Results

### 3.1. The Characteristics of the Jaundice Patients

The demographic and clinical characteristics of the 3610 patients with jaundice are shown in Table 1. Among the patients, 68.67% were male and approximately 50% were older than 45. The patients had an average age of 39.33 (median: 45). For the treatment, the average single dose was 5.66 g (median: 6), and the average duration was 6.62 days (median: 4).

### 3.2. Description and Analysis of the Liver Enzyme Indicators before and after the Medication

**Figure 1:**
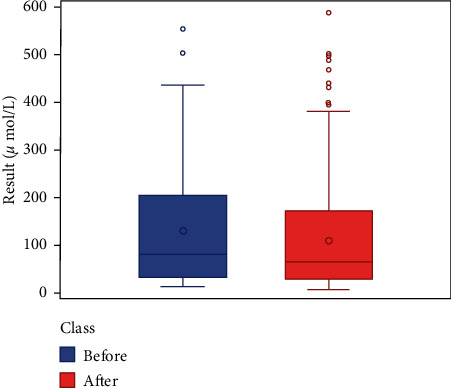
TBil boxplot (*n* = 369).

**Figure 2:**
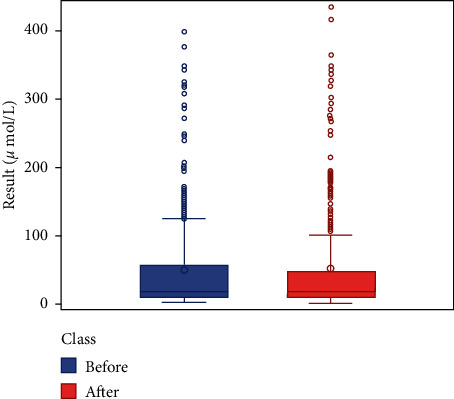
DBil boxplot (*n* = 392).

**Figure 3:**
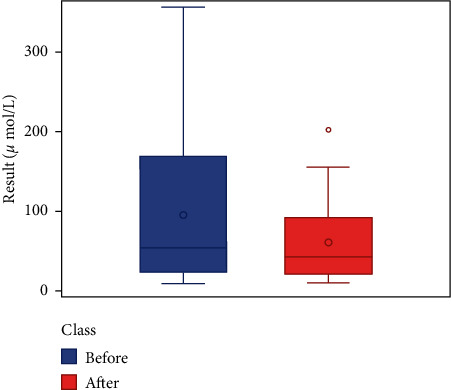
IBil boxplot (*n* = 49).

**Figure 4:**
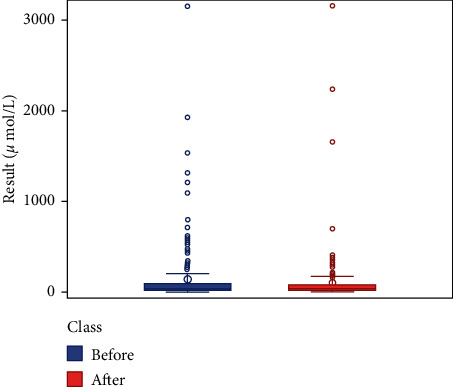
ALT boxplot (*n* = 166).

**Figure 5:**
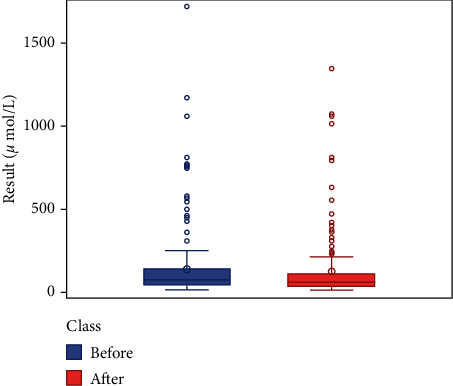
AST boxplot (*n* = 158).

**Figure 6:**
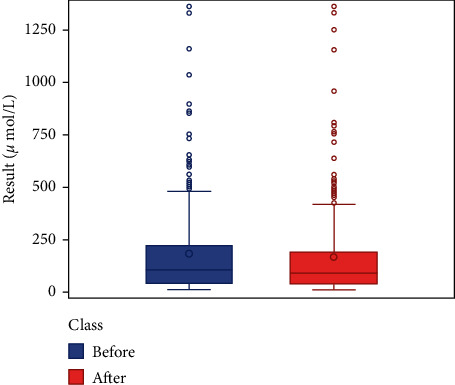
GGT boxplot (*n* = 228).

**Figure 7:**
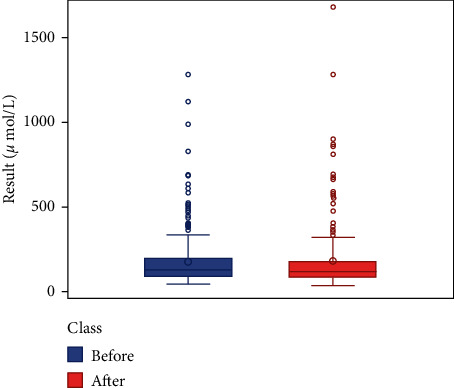
ALP boxplot (*n* = 231).

We respectively described and analyzed the general data of the jaundice patients' liver enzymes tested before medication (Table 2) and after medication (Table 3). We compared the jaundice patients' postmedication liver enzyme data boxplot with the premedication boxplot (Figures 1–7) and found that, while the average levels of TBil, IBil, AST, GGT, and ALP decreased after the medication, the average levels of DBil and ALT did not change significantly. In the figures, the middle line represents the average level of the liver enzyme analyzed.

### 3.3. Qualitative Analysis of Liver Enzyme Changes after the Medication

The liver enzyme changes in jaundice patients are presented in Tables 4 and 5, which show that, for most patients, the serum liver enzyme levels decreased after Yinzhihuang granule treatment. The patients with decreased IBil and TBil levels comprised the largest percentages, 61.22% and 56.64%, respectively, of the total patients. After Yinzhihuang granule treatment, the serum liver enzyme levels of most jaundice patients returned to normal. The patients with ALT and ALP levels normalized comprised the largest percentages, 81.93% and 79.22%, respectively, of the total patients.

### 3.4. Test of the Difference in Liver Enzyme Indicators before and after the Medication

Because the difference in liver enzyme indicators before and after the medication did not follow the normal distribution, we performed a nonparametric test known as Wilcoxon signed-rank, with the results presented in Table 6, from which one sees that the average TBil, IBil, GGT, and ALP levels decreased after Yinzhihuang granule treatment. Because *P* values were all less than 0.05, the differences were statistically significant, and the null hypothesis was rejected, demonstrating that the test results had a difference before and after the medication. TBil was an important indicator for diagnosing and judging the severity of jaundice, which can be used to estimate the curative effect. If TBil increased with the IBil increased significantly, it might be diagnosed as hemolytic jaundice [13]. The decrease of TBil and IBil indicated that Yinzhihuang granules can improve the degree of jaundice. The increase of GGT and ALP was more common in cholestatic jaundice [17]. The decrease of their values proved that cholestasis has been alleviated in patients with jaundice. After the Yinzhihuang granule treatment, the patients' average DBil level raised slightly and the average ALT level decreased, but both *P* values were greater than 0.05 (*P*=0.3801 and 0.0656, respectively), indicating that the differences were not statistically significant. After the Yinzhihuang granule treatment, the patients' average AST level markedly elevated. Considering that both sets of data were not normally distributed, the mean value may be inappropriate to describe the central tendency, and the use of the median value may be more reasonable. The postmedication median AST level of the jaundice patients was 29.00 U/L, which was lower than that before the medication (62.00 U/L). Because *P*=0.0278, the difference was statistically significant. The determination of ALT and AST showed that before the treatment of Yinzhihuang granules, the values of ALT and AST in patients with jaundice were 1.2 times higher than the normal upper limit, which reached the standard of diagnosis of liver injury [18]. After drug intervention, the values of ALT and AST decreased significantly, which showed that Yinzhihuang granules could reduce the degree of liver injury in patients with jaundice.

## 4. Discussion

### 4.1. Effects of Yinzhihuang Granules on Liver Enzyme Indicators in Jaundice Patients

TCM has a long history in treating jaundice. Huang Di Nei Jing stated that “when heat and dampness intersect, the people become sick.” According to Huang Di Nei Jing, the primary pathogenesis of jaundice is the accumulation of dampness and heat, which is caused by the pathogenic wind, which combines Yang with internal dampness, heat, and dampness accumulates in the spleen and spreads to the muscle surface. In treatment, it is necessary to remove dampness and yellow and dredge the viscera to relieve heat [19]. Zhongjing Zhang believes that the main cause of jaundice is spleen dampness and heat stasis, and the disease lies in Yangming Meridian, which is discussed in article 236: “Yangming disease, fever and sweating, this is heat, cannot yellowing. But the head is sweating, the body is not sweaty, the neck is close to the neck, and the urination is disadvantageous, and those who thirst for water are stagnant and hot, and the body will turn yellow. Yinchenhao decoction is the main one.” It is considered that jaundice caused by damp-heat should be treated with Yinchenhao decoction [20]. Yinzhihuang granules are derived from Yinchenhao decoction. The prescription consisting of four Chinese medicines (*Artemisiae Scopariae Herba*, *Scutellariae Radix*, *Gardeniae Fructus*, and *Lonicerae Japonicae*) [21] is used to treat jaundice caused by liver-gallbladder dampness-heat. The prescription retains the two main medicines in Yinchenhao decoction (*Artemisiae Scopariae Herba* and *Gardeniae Fructus*) with the strong purgative effect changed in *Scutellariae Radix* and *Lonicerae Japonicae*, which can slow down its purging ability and enhance its effect of clearing heat and removing dampness. According to TCM, the monarch drug *Artemisiae Scopariae Herba* is bitter, acrid, and slightly cold and has the effects of clearing damp-heat, increasing choleresis, and relieving jaundice [22]; the minister drug *Gardeniae Fructus* is bitter and cold and has the effects of purging fire, relieving restlessness, and clearing damp-heat [23]; *Scutellariae Radix* is bitter and cold and has the effects of clearing away heat and dry dampness, purging fire to eliminate toxin [24], and the compatibility of the three medicines strengthens the effect of clearing away dampness and heat; *Lonicerae Japonicae Flos* is cold and tastes sweet and has the effects of clearing heat, detoxifying, and anti-inflammation [25]. The four Chinese medicines work together compatibly to play the role of clearing heat and detoxifying and clearing dampness and removing jaundice. The clinical application of Yinzhihuang granules is mostly to treat neonatal jaundice [26] whose effectiveness has been verified in clearing jaundice and reducing enzyme activity. For example, some scholars demonstrated that Yinzhihuang granules, when combined with intermittent blue light irradiation, can reduce the bilirubin levels in jaundice neonates [27], the GGT levels, and the ALP levels; the differences were statistically significant (*P* < 0.05); the effectiveness and safety were both better than the sole blue light irradiation treatment [28].

The present study demonstrated that the jaundice patients' average serum TBil, IBil, AST, GGT, and ALP levels decreased after the Yinzhihuang granule treatment (*P* < 0.05), but their ALT levels were not significantly affected. Although the ALT levels decreased by 17.11 U/L, the difference was not statistically significant because *P*=0.0656. After the Yinzhihuang granule treatment, the average DBil level increased by 3.77 *μ*mol/L (*P* > 0.05). This elevation might be associated with the change of the conditions of the patients who had not yet recovered. These results, which were obtained by analyzing the existing real-world HIS data, demonstrate that Yinzhihuang granules help to reduce the jaundice patients' TBil (*P* < 0.0001), IBil (*P*=0.0017 < 0.05), AST (*P*=0.0278 < 0.05), GGT (*P*=0.0002 < 0.05), and ALP (*P*=0.0009 < 0.05) levels, but do not imply that Yinzhihuang granules help to reduce the jaundice patients' DBil (*P*=0.3801 > 0.05) and ALT (*P*=0.0656 > 0.05) levels.

The pharmacological mechanisms of Yinzhihuang granules have not yet been completely understood due to its complex active ingredients and versatile action pathways. Modern pharmacological studies have found that the volatile oil component contained in the monarch drug *Artemisiae Scopariae Herba* can obviously antagonize the serum ALT and AST activities induced by carbon tetrachloride (CCl4) in mice with liver injury and can thus protect the liver from CCl4 injury [29]. By inducing the liver enzyme system, *Artemisiae Scopariae Herba* enhances the liver's absorption, binding, and excretion of bilirubin and promotes the removal of bilirubin [30]. The minister drug *Gardeniae Fructus* may play an anticholestasis role by participating in biological processes such as the acute inflammatory response, positive regulation of reactive oxygen metabolism, and nitric oxide anabolism [31], thereby promoting bilirubin excretion [32]. In one study, Yinzhihuang granules were used to intervene estrogen-induced cholestasis in rats. After 14 days, it was found that the bile flow rate and the total bile flux increased, and that the levels of ALT, AST, ALP, TBil, DBil, IBil, and total bile acid (TBA) were significantly reduced [33]. The mechanism of removing jaundice and increasing choleresis may be the upregulation of the expression of multidrug resistance transporters Mrp2 and Mrp3 in the hepatocyte membrane and the enhancing of the excretion capability of hepatocytes with cholestasis [34], thereby reducing the accumulation of bilirubin and cholate in the hepatocytes [35]. Through network pharmacology studies, some scholars found that the target of Yinzhihuang granule-aided liver disease treatment may be the epidermal growth factor receptor (EGFR). Studies have confirmed that the EGFR plays an indispensable role in hepatocytes repair and regeneration and is a key regulator of the hepatocytes proliferation in the early stage of liver regeneration [36]. By promoting the proliferation of hepatocytes, EGFR plays a role in resisting liver damage [37]. There was a synergistic effect among the four traditional Chinese medicines of Yinzhihuang granules. Studies have confirmed that these four herbs can act directly on target proteins such as the androgen receptor, retinoic acid receptor *β*, and hepatocyte growth factor receptor, mainly by interfering with the phosphatidylinositol 3-kinase (phosphatidylcholine3-kinase, PI3K) signal pathway [37]. Among them, the hepatocyte growth factor was been proved to regulate the initiation of CD8^+^ T  cells in the hepatitis virus model, thus inhibiting liver inflammation and injury [38]. It was also an important biomarker for the diagnosis of biliary obstruction and liver injury in patients with obstructive jaundice [39].

Taken the present information together, Yinzhihuang granules have the following four mechanisms to remove jaundice, protect liver, and reduce enzyme activities: (1) preventing liver injury by antagonizing the serum ALT and AST activities induced by CCl4; (2) anticholestasis, promoting bilirubin excretion and clearance; (3) upregulating the expression of Mrp2 and Mrp3, reducing the accumulation of bilirubin; and (4) promoting the proliferation, repair, and regeneration of hepatocytes, thereby protecting the liver.

Although the above studies have beneficially explored from different angles, the mechanisms of Yinzhihuang granules in removing jaundice and reducing enzyme activities, more bioinformatics studies and experimental verifications are further needed to learn the mechanisms of multiingredient, multitarget, and multipathway drug action to remove jaundice and reduce enzyme activities, in consideration that the active ingredients of Yinzhihuang granules are complex and unclear, as well as the ingredient targets.

### 4.2. Real-World Research Based on the HIS Database

As a new research field, real-world studies have received ever-increasing attentions from the broad masses of doctors, researchers, and policy makers and have thus exerted profound influences upon the formulation of health policies. In 2010, Yanming Xie's research group published an article entitled “real-world research: a new idea for evaluating the effect of traditional Chinese medicine intervention measures,” which introduced the concept of real-world research to China for the first time [40]. After nearly a decade of development, China's real-world research has made progress in the fields of postmarketing evaluation of drugs, medical insurance decision-making, and medical equipment supervision. In the field of TCM, some scholars found that the primary applications of real-world research are the effectiveness and safety evaluation of the proprietary Chinese medicines after entering the market. Because 49.60% of the data were from HIS [41], HIS-based data analysis is the main mode of real-world research in the present field of TCM. The real-world evidences obtained by the HIS-based analysis make up to a certain degree the extrapolation limits of randomized controlled trial conclusions and can better evaluate the actual therapeutic effect and safety of a proprietary Chinese medicine in the practical medical environment [42]. Although the efficacy of Yinzhihuang granules in treating jaundice has been confirmed by a number of randomized controlled trials, the questions have not been answered by relevant research studies such as the actual effects of clinical application and the influence upon the liver enzyme indicators. By extracting a huge amount of data from many 3A hospitals in China, a large-scale electronic medical record integration data warehouse was established by the Institute of Basic Research in Clinical Medicine, China Academy of Chinese Medical Science [43]. Through processes such as data cleaning and standardization, the analyzable real-world data were finally formed, which laid the groundwork for the evaluation of the safety and real-world effectiveness of Yinzhihuang granules after entering into the market. Through the analysis of HIS data by the descriptive statistical method and nonparametric test (Wilcoxon sign-ranked), we obtained the actual intervention effect of Yinzhihuang granules on the serum liver enzymes of jaundice patients in the real medical environment, and the results were consistent with the relevant literature. The real-world evidence obtained in this study can supplement to some degree the evidence chain of the effectiveness evaluation of Yinzhihuang granules after entering into the market and provide reference and basis for further clinical trials and research studies on pharmacological mechanisms.

However, HIS data-based real-world studies are limited by data missing and confounding factors, which reduce to some degree the accuracy of causal inference. This study only described the changes of serum liver enzyme indicators in jaundice patients, without considering the other factors that could affect the curative effect (e.g., the use of other drugs to treat jaundice may decrease the levels of serum liver enzymes); thus, the causal inference of therapeutic outcomes was not very convincing. Randomized controlled trials remained the primary means of efficacy evaluation. For the next step, the results should be verified by carrying out multicenter, randomized, double-blind, controlled trials that are of high-quality and prospective; the pharmacological mechanisms also need to be further studied. Moreover, this study was not able to evaluate the safety of treating jaundice patients with Yinzhihuang granules due to the lack of records of adverse reactions and the fact that the patients were not followed up for a certain period. Prospective, multicenter, safe hospital monitoring should also be the focus of the next step research.

## 5. Conclusions

Based on the existing real-world HIS data analysis, we found that Yinzhihuang granules help to decrease jaundice patients' TBil, IBil, AST, GGT, and ALP levels; there are no evidence of Yinzhihuang granules decreasing jaundice patients' DBil and ALT levels.

## Figures and Tables

**Table 1:** The demographic and clinical characteristics of the jaundice patients.

Characteristic	Population
Gender	
Male	2479
Female	1087
Missing	44
Age	
≤12 yr	760
12–18 yr	54
18–45 yr	1029
45–65 yr	1242
65–85 yr	507
>85 yr	16
Hospitalization time	
≤3 d	242
3–7 d	491
7–14 d	831
14–28 d	1065
Missing	67
Western medicine diagnosis^a^	
Neonatal hyperbilirubinemia	238
Pulmonary tuberculosis	170
Chronic active hepatitis B	79
TCM syndrome^b^	
Damp-heat brewing internally	67
Deficiency of both qi and blood	32
Yang qi deficiency	32
Single dose	
≤6 g	3337
6–12 g	47
12–18 g	16
18–30 g	37
30–60 g	23
>60 g	20
Missing	6
Course of treatment	
≤3 d	1269
4–7 d	567
8–14 d	530
15–28 d	432
Missing	649
Combined use of drugs^c^	
TCM: Ganmao Qingre granules	416
TCM: Kuhuang injection	382
TCM: Bupleurum injection	360
Western medicine: glycyrrhizic acid	1831
Western medicine: thymosin	1028
Western medicine: dexamethasone	592

^a,b,c^The top three in population.

**Table 2:** Description of premedication serum liver enzyme indicators (nonnormal distribution)^*∗*^.

Variable	Number of case (*n)*	Median	Lower quartile	Interquartile range	Upper quartile	Minimum	Maximum
TBil (*μ*mol/L)	952	79.00	35.50	169.80	205.30	12.00	609.60
DBil (*μ*mol/L)	1027	16.90	9.00	37.00	46.00	0.10	402.90
IBil (*μ*mol/L)	241	66.10	27.20	134.40	161.60	8.50	379.30
ALT (U/L)	596	25.00	13.00	50.50	63.50	4.00	3160.0
AST (U/L)	586	62.00	38.00	83.00	121.00	6.00	2232.0
GGT (U/L)	706	105.00	43.00	181.00	224.00	4.00	2487.0
ALP (U/L)	732	143.50	100.00	107.50	207.50	43.60	2098.0

^*∗*^The number of cases (*n*) counts the patients who had a premedication test.

**Table 3:** Description of the postmedication serum liver enzyme indicators (nonnormal distribution)^*∗*^.

Variable		Number of case (*n)*	Median	Lower quartile	Interquartile range	Upper quartile	Minimum	Maximum
TBil (*μ*mol/L)		549	69.10	28.20	146.20	174.40	5.70	766.60
DBil (*μ*mol/L)		550	14.75	8.00	30.50	38.50	0.30	560.20
IBil (*μ*mol/L)		62	43.55	19.80	72.90	92.70	10.10	289.90
ALT (U/L)		245	27.00	14.00	57.00	71.00	3.00	3160.0
AST (U/L)		225	29.00	35.00	82.00	117.00	15.00	13750.0
GGT (U/L)	357	94.00	43.00	173.00	216.00	9.00	3645.00
ALP (U/L)	349	124.00	91.00	97.00	188.00	15.00	1683.00

^*∗*^The number of cases (*n*) counts the patients who had a postmedication test.

**Table 4:** Changes of serum liver enzyme indicators after the medication (decrease/increase).

Variable	Decrease/counts	Increase/counts	Invariance/counts	Total
TBil (*μ*mol/L)	209 (56.64%)	88 (23.85%)	72 (19.51%)	369 (100%)
DBil (*μ*mol/L)	177 (45.15%)	13 (35.20%)	77 (19.64%)	392 (100%)
IBil (*μ*mol/L)	30 (61.22%)	16 (32.65%)	3 (6.12%)	49 (100%)
ALT (U/L)	78 (46.99%)	60 (36.14%)	28 (16.87%)	166 (100%)
AST (U/L)	84 (53.16%)	54 (34.18%)	20 (12.66%)	158 (100%)
GGT (U/L)	122 (53.51%)	73 (32.02%)	33 (14.47%)	228 (100%)
ALP (U/L)	119 (51.52%)	79 (34.20%)	33 (14.29)	231 (100%)

**Table 5:** Classification of serum liver enzyme indicators after the medication (normal/abnormal).

Variable	Normal/counts	Abnormal/counts	Total
TBil (*μ*mol/L)	281 (76.15％)	88 (23.85%)	369 (100%)
DBil (*μ*mol/L)	268 (68.37%)	124 (31.63%)	392 (100%)
IBil (*μ*mol/L)	35 (71.43%)	14 (28.57%)	49 (100%)
ALT (U/L)	136 (81.93%)	30 (18.07%)	166 (100%)
AST (U/L)	114 (72.15%)	44 (27.85%)	158 (100%)
GGT (U/L)	177 (77.63%)	51 (22.37%)	228 (100%)
ALP (U/L)	183 (79.22%)	48 (20.78%)	231 (100%)

**Table 6:** Difference in the serum liver enzyme levels before and after the medication.

Variable	Number of case (*n)*	Premedication (mean ± SD)	Postmedication (mean ± SD)	Statistics (*S)*	*P* value
TBil (*μ*mol/L)	369	126.46 ± 109.82	112.42 ± 109.64	9106.50	≤0.001
DBil (*μ*mol/L)	392	43.19 ± 62.70	46.96 ± 79.43	1422.50	0.3801
IBil (*μ*mol/L)	49	96.22 ± 82.22	63.03 ± 53.66	277.00	0.0017
ALT (U/L)	166	108.87 ± 295.46	91.76 ± 282.56	865.50	0.0656
AST (U/L)	158	135.55 ± 241.51 Median = 62.00	200.28 ± 986.27 Median = 29.00	1031.50	0.0278
GGT (U/L)	228	190.13 ± 250.46	182.61 ± 288.87	2872.50	≤0.001
ALP (U/L)	231	187.77 ± 163.00	186.43 ± 197.09	2656.50	≤0.001

## Data Availability

The datasets used and/or analyzed during the current study are available from the corresponding author upon request.
